# Sepsis Associated Delirium

**DOI:** 10.3390/medicina56050240

**Published:** 2020-05-18

**Authors:** Ben Atterton, Maria Carolina Paulino, Pedro Povoa, Ignacio Martin-Loeches

**Affiliations:** 1 Department of Intensive Care Medicine, Multidisciplinary Intensive Care Research Organization (MICRO), St. James’s Hospital, St. James Street, Dublin 8, Dublin, D08 NHY1, Ireland; ben.atterton3@gmail.com; 2 Polyvalent Intensive Care Unit, São Francisco Xavier Hospital, Centro Hospitalar de Lisboa Ocidental, 1449-005 Lisbon, Portugal; mcarolinapaulino@gmail.com (M.C.P.); pedrorpovoa@gmail.com (P.P.); 3 NOVA Medical School, CHRC, New University of Lisbon, 1099-085 Lisbon, Portugal; 4 Center for Clinical Epidemiology and Research Unit of Clinical Epidemiology, OUH Odense University Hospital, 5000 Odense, Denmark; 5 Hospital Clinic, IDIBAPS, Universidad de Barcelona, Ciberes, 08036 Barcelona, Spain

**Keywords:** sepsis, delirium, ICU, dexmedetomidine

## Abstract

Sepsis is a potentially life-threatening condition caused by a systemic dysregulated host response to infection. The brain is particularly susceptible to the effects of sepsis with clinical manifestations ranging from mild confusion to a deep comatose state. Sepsis-associated delirium (SAD) is a cerebral manifestation commonly occurring in patients with sepsis and is thought to occur due to a combination of neuroinflammation and disturbances in cerebral perfusion, the blood brain barrier (BBB) and neurotransmission. The neurological impairment associated with SAD can persist for months or even longer, after the initial septic episode has subsided which may impair the rehabilitation potential of sepsis survivors. Early identification and treatment of the underlying sepsis is key in the management of SAD as once present it can be difficult to control. Through the regular use of validated screening tools for delirium, cases of SAD can be identified early; this allows potentially aggravating factors to be addressed promptly. The usefulness of biomarkers, neuroimaging and electroencephalopathy (EEG) in the diagnosis of SAD remains controversial. The Society of Critical Care Medicine (SCCM) guidelines advise against the use of medications to treat delirium unless distressing symptoms are present or it is hindering the patient’s ability to wean from organ support.

## 1. Introduction

Sepsis is a leading cause of morbidity and mortality around the world. It is a systemic, dysregulated, inflammatory reaction to an infection and can have profound effects on all organ systems which if left untreated often leads to multi-organ failure and death. The delicately balanced central nervous system is particularly susceptible to dysfunction however the mechanisms through which sepsis affects the brain are poorly understood and often underappreciated. Between a quarter and a third of septic patients show signs of neurological involvement including confusion, agitation and coma or “sepsis-associated delirium” (SAD). In this review article we will discuss the currently understood pathophysiology, diagnostic tools and management strategies for patients with SAD as well as potential future treatment options.

## 2. Pathophysiology

There is still much to learn about the pathophysiology of SAD, however it is currently understood to be a combination of neuroinflammation and disturbances in cerebral perfusion, the blood brain barrier (BBB) and neurotransmission. Post-mortem studies of septic patients show cerebral haemorrhage, ischaemia, multifocal necrotising leukoencephalopathy, micro-abscesses and neuronal apoptosis [1,2,3,4].

### 2.1. Endothelial Dysfunction and Cerebral Perfusion

Endothelium forms a functional component of the BBB; its expression of adhesion molecules, production of nitric oxide (NO), signalling pathways and overall cellular integrity are affected by inflammatory cytokines, which are markedly elevated in sepsis [5]. Impaired peripheral vascular reactivity, a marker of endothelial dysfunction, is associated with fewer delirium-free days in septic patients [5]. It stands to reason therefore that endothelial dysfunction plays a role in SAD, possibly due to altered cerebral perfusion and BBB permeability [5].

Several studies have shown, by means of transcranial-doppler, that cerebrovascular autoregulation is impaired in patients with SAD [6,7,8]. This failure of the brain to autoregulate blood flow renders the septic patient more vulnerable to extremes of blood pressure. Severe hypotension is associated with SAD [2], likewise hypertension above the autoregulatory range has also been implicated in the development of post-operative delirium [9]. Whilst hypertension is less common than hypotension in severe sepsis, it can occur if close attention to vasoactive medications is not maintained. This begs the question of whether individualised mean arterial pressure (MAP) targets derived from patients’ own autoregulation ranges should be determined early in sepsis to maintain consistent cerebral perfusion in an effort to prevent SAD.

### 2.2. Neurotransmission

A broad range of signalling molecules are involved in the pathophysiology of SAD including neuropeptides and neurotransmitters such as acetylcholine, γ-aminobutyric acid (GABA), noradrenaline, dopamine and serotonin.

The cholinergic nervous system has a role in levels of arousal and higher cognitive functions such as learning and memory. These functions are characteristically deranged in delirium and it is postulated that a hypoactive cholinergic system leads to changes in cognition, as demonstrated by patients with dementia or those treated with anti-cholinergic drugs. The exact role of acetylcholine in SAD has yet to be fully elucidated but limited evidence from animal models suggests that exposure to a septic stimulus results in reduced cerebral cholinergic activity [10].

Various studies have found abnormal levels of neurotransmitter precursors, including amino acids, in both the serum and cerebrospinal fluid of patients with SAD [11,12]. The significance of this is unclear but may suggest concurrently deranged levels of neurotransmitters in sepsis and abnormal neurotransmission, however it may also reflect protective mechanisms to detoxify phenylalanine levels [13].

A complex interplay between neuropeptides such as substance P, oxytocin, cortisol, orexin and melatonin is involved in the regulation of vegetative functions such as sleep, feeding behaviour and energy homeostasis [14]. Sepsis results in deranged levels of these signalling molecules and thus it seems logical that they are involved in SAD [14].

### 2.3. Microglial Activation

Sepsis induces the activation of microglial cells with subsequent oxidative damage to the BBB and an increase in pro-inflammatory cytokines such as tumour necrosis factor-α (TNFα) and the interleukins IL-1β and IL-6 [15,16]. The neuroinflammation that ensues can persist for months after the initial septic episode has subsided and lead to demonstrable permanent neuronal loss; this may explain the longer-term neurological decline that is often seen in sepsis survivors [17]. Microglial activation and the cytokine surge are amplified in aging brains; this is observed clinically in the propensity of older patients to develop delirium despite seemingly innocuous stimuli [18,19]. When microglia have been inhibited in rats their cognitive function is preserved following a septic episode suggesting that microglial overactivation may play a crucial role in the development of SAD [15,19]. Microglial activation can be attenuated by vagal nerve stimulation due to their expression of nicotinic receptors, the activation of which by acetylcholine slows their pro-inflammatory activities [16]; however as mentioned above the cholinergic nervous system is often hypoactive in delirium. 

## 3. Diagnosis

Multiple studies have found that ICU clinicians often fail to recognise delirium without the use of screening tools [20]. The most recent clinical practice guidelines on delirium from the Society of Critical Care Medicine (SCCM) recommend regularly assessing for delirium using a validated tool such as the Confusion Assessment Method-ICU (CAM-ICU) or the Intensive Care Delirium Screening Checklist (ICDSC) [21,22,23,24]. It has been shown that CAM-ICU demonstrates a higher sensitivity (80%) and specificity (95.9%) than the ICDSC (sensitivity 74% and specificity 81.9%) [25], which may account for its more widespread use. A recent publication describes a new validated tool, CAM-ICU-7, which not only helps to identify delirium but also allows assessment of the severity of the delirium with more severe forms being associated with worse outcomes [26]. Bedside screening tests need to be performed regularly as otherwise they may underestimate the prevalence of delirium by failing to take into account the fluctuating nature of delirium.

Neuroimaging can be used to study structural and functional brain abnormalities associated with delirium as well as identifying risk factors including structural abnormalities (i.e. periventricular white matter disease and atrophy), incipient dementia, amyloid deposition and cholinergic dysfunction [27,28,29]. The most common structural abnormalities found in the delirious brain are atrophy and impaired white matter integrity (including white matter hyperintensities) whilst ischaemic lesions, oedema and areas of inflammation have also been identified [30,31]. Abnormalities are frequently found in the frontal lobe and limbic system, as well as the parietal and temporal lobes [32]. These alterations persist for 3–5 months after discharge, further highlighting the possible link between delirium and long-term cognitive impairment [33,34]. Most of these studies however, had small sample sizes, poor study design, variation in imaging methods, inappropriate or questionable delirium measurements and failed to consider confounding variables so further work is needed [35].

It has been demonstrated that biomarkers have limited clinical utility in diagnosing delirium or predicting its duration and severity [36]. One of the biggest reviews on this subject included 32 studies reporting information on 7610 patients aged 60 and older. They concluded that the use of biomarkers to identify delirium was not recommended [36]. Routinely used inflammatory biomarkers and those of brain-specific metabolism have been widely studied in delirium. Recent work correlated markers of systemic inflammation and those of astrocyte and glial cell activation (IL-6, IL-8, IL-10, TNF-α, C-reactive protein and S-100β levels) with longer duration of delirium, more severe delirium and higher in-hospital mortality [37]. It was shown that higher IL-8 and S-100β levels were associated with increased mortality among delirious patients [26]. These results highlight the pathophysiological role of inflammation and astrocyte activation in delirium, namely in its duration and severity. The development of delirium is not preceded by a change in the profile of inflammatory biomarkers or brain proteins; this is a major limitation in their usefulness as they cannot be used to predict or identify those at risk of delirium [38].

The use of conventional electroencephalography (EEG) in the diagnosis and monitoring of delirium is well established [39,40]. Following the work of Jacobson et al. we have begun to understand the pattern of EEG changes in delirious patients, namely an increased slow-wave activity and a slowed and disrupted alpha rhythm [41]. This generalized slowing on routine clinical EEG strongly correlates with delirium and may be a valuable marker of delirium severity [42]. Generalized EEG slowing also provides some prognostic information as the degree of slowing correlates with overall delirium severity, worse clinical outcomes, increased length of stay, worse Glasgow Outcome Scale and increased mortality [42]. Despite the demonstrable value of EEG in diagnosis of delirium, it is not suitable for screening due its size, cost and the expertise required for lead placement and interpretation. As with the existing bedside screening tools mentioned above, routine EEG does not reliably assess the fluctuating course of delirium. To that end, Nielsen et al., studied the relevance of continuous EEG (cEEG) to aid in the diagnosis of delirium in septic patients [43]. They concluded that delirious episodes were associated with the disappearance of high-frequency electrographic cEEG activity (beta waves) and the increased power of low-frequency activity (delta waves) [43]. Preserved cEEG power in the beta band was the strongest predictor of the absence of delirium in awake or lightly sedated ICU patients with sepsis [43].

## 4. Subsyndromal Delirium

Subsyndromal delirium (SSD) is common but often poorly recognized in the ICU. It is usually characterized as a milder cognitive dysfunction, sometimes interpreted as an intermediate stage between delirium and a normal mental state [44]. To date there is no consensus on the exact definition or diagnosis of SSD, however the Diagnostic and Statistical Manual of Mental Disorders, 5th Edition (DSM-V), defines the concept of an “attenuated delirium syndrome” which seems to describe a condition very similar to what we consider SSD but without this label [45].

The ICDSC screening tool includes SSD as a specific diagnosis and can therefore be used to aid in the diagnosis. An ICDSC score between 1 and 3 (out of 8 items) corresponds to SSD. Later, the CAM-ICU was also adapted to include SSD, with the presence of one positive item out of the 4 suggesting a diagnosis of SSD [46].

Only two studies have evaluated the relationship between SSD and mortality. Ouimet el al. reported an increase in ICU mortality in the SSD group compared to patients without delirium but after adjusting for age, APACHE II score and coma-inducing medication there was no statistically significant difference between the groups [47]. Breu et al. showed that hospital mortality among patients with SSD and without delirium is comparable [48].

According to the most recent meta-analysis, which included 2630 patients, SSD was present in 950 patients (36%) [49]. The study demonstrated a relationship between SSD and increased length of hospital stay but showed no association with mortality [49]. Another study, not included in this meta-analysis, revealed a relationship between the duration of SSD, as diagnosed by CAM-ICU and the requirement for long-term care [24].

Although the progression of SSD to delirium has not been proven, some studies have trialled the use of antipsychotics in the prevention of delirium in patients with SSD. Al-Qagheeb et al. used antipsychotics (haloperidol 1 mg versus placebo every 6 hours) in 60 mechanically ventilated patients but were unable to prevent progression of SSD to delirium, duration of delirium or time to first delirious episode [50]. Hakim et al. demonstrated that administration of risperidone to patients with SSD following cardiac surgery, was associated with a significant reduction in the occurrence of delirium [51]. This, however, is the only study demonstrating such a relationship between the two entities and the pharmacological prevention of conversion to delirium. Current SCCM guidelines do not recommend administration of drugs for SSD treatment [21].

An exact definition of SSD, either as its own distinct pathological entity or as a milder form of delirium, is required, as are further studies into diagnostic tools and management.

## 5. Treatment

**Figure 1:**
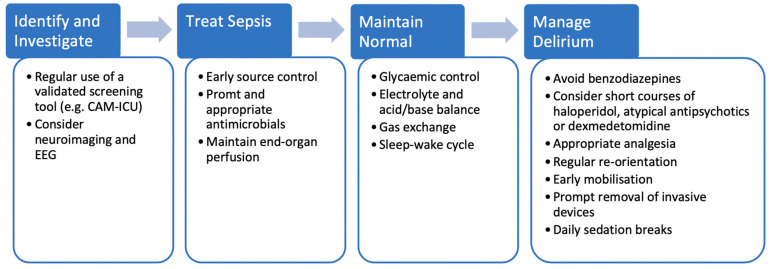
Investigation and Management of sepsis-associated delirium (SAD).

There is currently no specific treatment for SAD and it remains the case that early identification and management of the underlying sepsis provides the patient with the best chance of avoiding cognitive sequelae (Figure 1). The latest iteration of the Surviving Sepsis Campaign guideline advocates the use of early source control, prompt administration of appropriate antimicrobials and the maintenance of end-organ perfusion however they do not specifically mention SAD or its management [52]. A 2017 retrospective analysis of 2513 ICU patients, of which 53% had SAD, revealed that the most common modifiable factors at admission to ICU associated with the development of SAD were acute renal failure, abnormal blood glucose (both hyper- and hypoglycaemia), hypercapnoea and hypernatraemia [53]. The authors themselves acknowledge that many of these factors can, on their own, lead to altered cognition and that a causal relationship with SAD cannot be drawn [53] but maintenance of normal is arguably a cornerstone of intensive care medicine and so appropriate glycaemic control, correction of electrolytes and acid-base balance and so forth, are of fundamental importance when managing patients with SAD. 

Poor sleep contributes to the development of delirium, impairs the immune system and results in increased mortality and ICU length of stay [54]. Measures, therefore, should be taken to optimise patients’ sleep where possible. These include minimising nocturnal interventions and noise, the use of melatonin and carefully considered timing of medications, for example corticosteroids. One study looking at the use of nocturnal earplugs showed a reduction in the incidence of mild delirium in ICU patients [55].

The use of medications such as haloperidol for prophylaxis against delirium have not been shown to be of any benefit [56]. Although there is a postulated role of acetylcholine in SAD and despite their proven use in dementias, the routine use of cholinesterase inhibitors have also not been shown to reduce the severity or incidence of delirium in critically unwell adults [57]. Indeed the 2018 clinical practice guidelines on delirium from the SCCM do not recommend the use of any medications for the prevention of delirium due to a lack of statistical significance or meaningful outcomes amongst the literature [21].

Other established methods of preventing delirium in ICU patients include appropriate analgesia, effective and regular communication and re-orientation of the patient, restoration of hearing aids and glasses if required, early mobilisation and the prompt removal of redundant invasive devices [21,58,59]. Whilst not specific to SAD these are simple and, for the most part, innocuous interventions which should be encouraged.

Overwhelmingly the evidence points away from the use of benzodiazepines in delirium as they themselves have been found to be an independent cause of delirium [60]. Over a decade ago the MENDS trial found that patients had significantly more delirium free days when sedated with dexmedetomidine vs. lorazepam [61]. This paper was not specific to patients with sepsis however the majority of their patients were septic at admission and an a priori analysis of this subgroup revealed an even more pronounced benefit when compared to non-septic patients [62]. Similar beneficial effects of dexmedetomidine including improved patient interaction and communication have been found when compared to midazolam, clonidine, haloperidol, propofol and placebo [21,63,64,65,66].

The SCCM guidelines advise against the use of medications to treat delirium unless distressing symptoms are present or it is hindering the patient’s ability to wean from organ support. In these situations, they recommend the short-term use of either haloperidol, dexmedetomidine or an atypical antipsychotics such as quetiapine [21]. They do, however, specifically warn of the risk of patients discharged from the ICU ending up on unnecessarily prolonged and detrimental courses of these medications, they should therefore be stopped as soon as they are no longer required [21]. Other important considerations when using sedative agents are maintaining an appropriate level of sedation based on validated scales such as the Richmond Agitation Sedation Scale (RASS) and encouraging a daily break from continuous sedation to allow patients to be reoriented [21,67].

**Figure 2:**
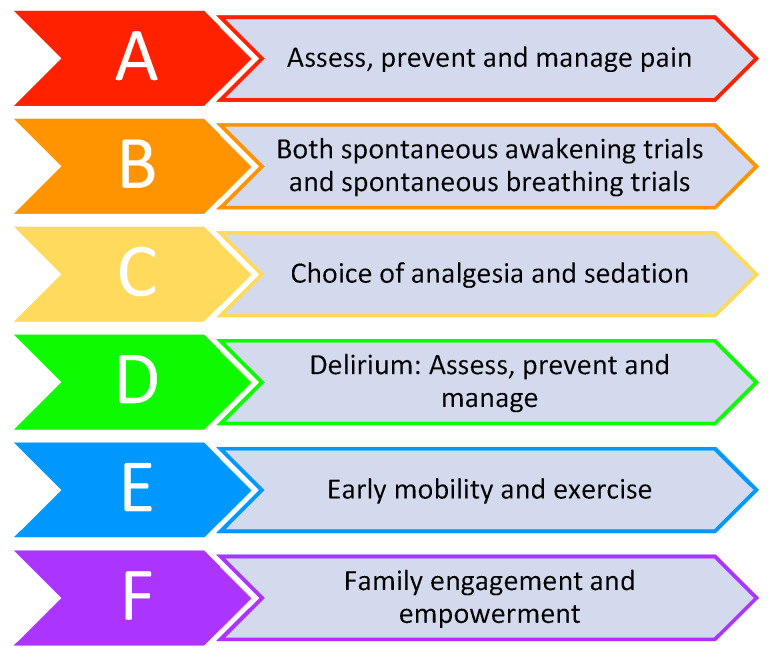
ABCDEF bundle.

The length of time patients spend in a delirious state during their acute illness has consistently been demonstrated to be a risk factor for the development of longer-term cognitive impairment [68,69,70]. Every effort should therefore be made to limit the duration of cognitive insult to patients during the early stages of their illness to minimise long term sequalae. The use of standardised models of care such as the ABCDEF bundle (see Figure 2), which incorporates many of the previously mentioned steps, have shown promising patient-centred results and as such are gaining popularity around the world [71,72].

## 6. Conclusions

Sepsis-associated delirium is a cerebral manifestation commonly occurring in patients with other infection-related organ dysfunctions and is caused by a combination of neuroinflammation and disturbances in cerebral perfusion. The use of validated assessment tools and EEG can help identify patients with SAD however the use of biomarkers remains unproven. Unfortunately, there is no specific treatment for SAD and it remains the case that early identification and management of the underlying sepsis coupled with the targeted use of sedatives drugs and regular re-orientation exercises is the most effective way of managing patients with SAD.
